# Querying a Clinical Data Warehouse for Combinations of Clinical and Imaging Data

**DOI:** 10.1007/s10278-022-00727-3

**Published:** 2022-11-23

**Authors:** Mathias Kaspar, Leon Liman, Caroline Morbach, Georg Dietrich, Lea Katharina Seidlmayer, Frank Puppe, Stefan Störk

**Affiliations:** 1 Department of Health Services Research, Carl Von Ossietzky University of Oldenburg, Campus Haarentor, V4/1/129, Ammerländer Heerstraße 140, 26129 Oldenburg, Germany; 2 Comprehensive Heart Failure Center and Department of Internal Medicine I, University and University Hospital Würzburg, Würzburg, Germany; 3 Chair of Computer Science VI, University of Würzburg, Würzburg, Germany; 4 Department of Cardiology, University Hospital Oldenburg, Oldenburg, Germany

**Keywords:** Clinical data warehouse, Medical images, System integration, Secondary data usage

## Abstract

This study aims to show the feasibility and benefit of single queries in a research data warehouse combining data from a hospital’s clinical and imaging systems. We used a comprehensive integration of a production picture archiving and communication system (PACS) with a clinical data warehouse (CDW) for research to create a system that allows data from both domains to be queried jointly with a single query. To achieve this, we mapped the DICOM information model to the extended entity–attribute–value (EAV) data model of a CDW, which allows data linkage and query constraints on multiple levels: the patient, the encounter, a document, and a group level. Accordingly, we have integrated DICOM metadata directly into CDW and linked it to existing clinical data. We included data collected in 2016 and 2017 from the Department of Internal Medicine in this analysis for two query inquiries from researchers targeting research about a disease and in radiology. We obtained quantitative information about the current availability of combinations of clinical and imaging data using a single multilevel query compiled for each query inquiry. We compared these multilevel query results to results that linked data at a single level, resulting in a quantitative representation of results that was up to 112% and 573% higher. An EAV data model can be extended to store data from clinical systems and PACS on multiple levels to enable combined querying with a single query to quickly display actual frequency data.

## Introduction

Clinical data warehouses (CDW) enable quick queries on homogenized data of a large number of patients and data of a multitude of clinical subsystems as shown by many examples [1–5]. CDWs can be used for a variety of reasons, including rapid feasibility testing or long-term data processing support for individual studies [6, 7].

Medical imaging data, unlike the types of data commonly documented in CDWs (i.e., numeric, categorical, and textual data from various clinical subsystems), are less frequently integrated into CDWs and tend to be more segregated from them [8, 9]. Medical imaging data has distinct features that render its use in CDWs more challenging. In particular, its pixel-based information results in much larger data sizes, which impedes its simple pseudonymized duplication into a CDW and hence its immanent usability. The increasingly better-defined analytic strategies that are supported by deep learning and artificial intelligence render the combination of clinical and imaging data a highly attractive research and development area [9–11]. But not only can the search for information in the pixel data itself be useful for this, but also the enhanced search in DICOM metadata [12, 13].

We previously showed the overall feasibility of a comprehensive integration architecture of a production PACS (including identified data) to a research CDW (including pseudonymized data) using ad hoc pseudonymization [14]. The most comprehensive PACS-CDW integration of the related work so far has been shown for i2b2, which primarily requires selecting a patient population in the CDW using clinical data that then becomes the basis for a PACS query in another downstream module [15]. Other CDW systems often only connect to a dedicated research PACS including image data of patient subgroups [16–22], a CDW dedicated to a specific disease [23] or are rather specialized for imaging analysis and less on clinical data [12, 24–28].

CDWs are often based on an entity–attribute–value (EAV) data schema (e.g., i2b2 [4]), a single data model into which data from various source systems (e.g., clinical subsystems) and their data models (e.g., the data models of specific structured forms) must be integrated. A common method used to provide data in EAV-based CDWs is an early aggregation of single or multiple values of different data models into individually usable variables. Such variables can typically be searched on the level of a patient or encounter, e.g., search for encounters with a laboratory NT-proBNP value > 1000 pg/ml and an ICD^1^ code = “I50” to detect patient encounters with heart failure.

However, some queries require a more detailed integration of subsets of the original data model to allow comparing parameters at the level of a document or more detailed groupings, e.g., search for patient encounters with elevated troponin T and elevated creatine kinase-MB (CK-MB) levels in the same laboratory report to detect patients with an ST elevation myocardial infarction (STEMI). Such a search requires integration of individual data elements linked not only via the patient and encounter identifier, but also via a document identifier (i.e., linkage of laboratory values to the laboratory report). The integration of a structured form containing further groupings (e.g., a table) would require an even more detailed linkage. A detailed example is shown in the online supplement.

Radiology datasets often consist of narrative radiology reports and associated imaging data that is structured in the multilevel DICOM data model. A detailed search for combinations of clinical and imaging data could also benefit from data integration at multiple grouping levels, i.e., to search for specific DICOM series instead of just discovering whether or not a patient or case has assigned images. However, we have not found another CDW that offers the selection and search on the document and further detailed levels for imaging metadata. Thus, querying and extracting related data from PACS and other clinical subsystems for research on large numbers of patients is impeded by limited integration into CDWs and manual downstream processing steps.

## Objectives

The objective of this study is to show the feasibility and benefit of single queries in a research data warehouse combining data from a hospital’s clinical and imaging systems.

## Methods

### Evaluation Methodology

We first contrast the data models commonly used in the clinical and imaging domain. Then, we describe the CDW used, its abstract data model, and the PACS middleware we used for the DICOM data integration. This basis is used to describe the method we used to integrate the DICOM data model into the CDW’s abstract data model, by preserving the linkages of the DICOM data model as far as possible. Since the existing generic CDW query interface was only able to query variables on either of the patient, encounter, or document level, we extended the PACS-CDW-middleware with a simple graphical query interface optimized for the combined query of imaging and clinical data.

Finally, we demonstrate the viability of a combined query using data entries from clinical and imaging systems using multiple grouping levels. Therefore, we selected the following two real inquiries from researchers, both of which mandate combinations of clinical and imaging data but different query approaches:

1. Radiology-oriented: Retrieve combinations of a radiology report and associated DICOM series of cardiac magnetic resonance imaging (MRI).
2. Disease-oriented: Detect patients with heart failure and retrieve combinations of the radiology report and associated MRI DICOM series

Whereas, the first inquiry has its focus rather on identifying DICOM series with similar image acquisition characteristics in combination with a radiology report, the second has its focus on retrieving DICOM series and clinical data for a specific disease. Both inquiries had a similar goal of providing potentially large amounts of data to researchers who planned a data analysis using deep learning approaches. However, in this work, we only focus on the optimized data retrieval in contrast to an overall retrieval and analysis pipeline. In addition, we chose very simple queries to show the benefit of a multilevel query, as opposed to more complex queries, as e.g., including DICOM tags that are documented only by special request from the manufacturer of a research MRI.

A single query was defined for each inquiry and executed using the new query interface. Resulting SQL queries were analyzed using the CDW’s relational database. Extracted query results were characterized using descriptive statistics. To show the benefit of a multilevel query, the query results were compared with the results of queries performed only on individual grouping levels (e.g., document or encounter level) in order to simulate systems that do not support multiple grouping levels. We then discuss these results with existing and potentially future integration into a very similar EAV-based CDW and a refined relational data model.

### Medical Data

From a data homogenization perspective when using a CDW, the data models of most clinical subsystems are fairly similar, but differ regarding the data model employed in PACS.

#### Clinical Data Model

**Fig. 1:**
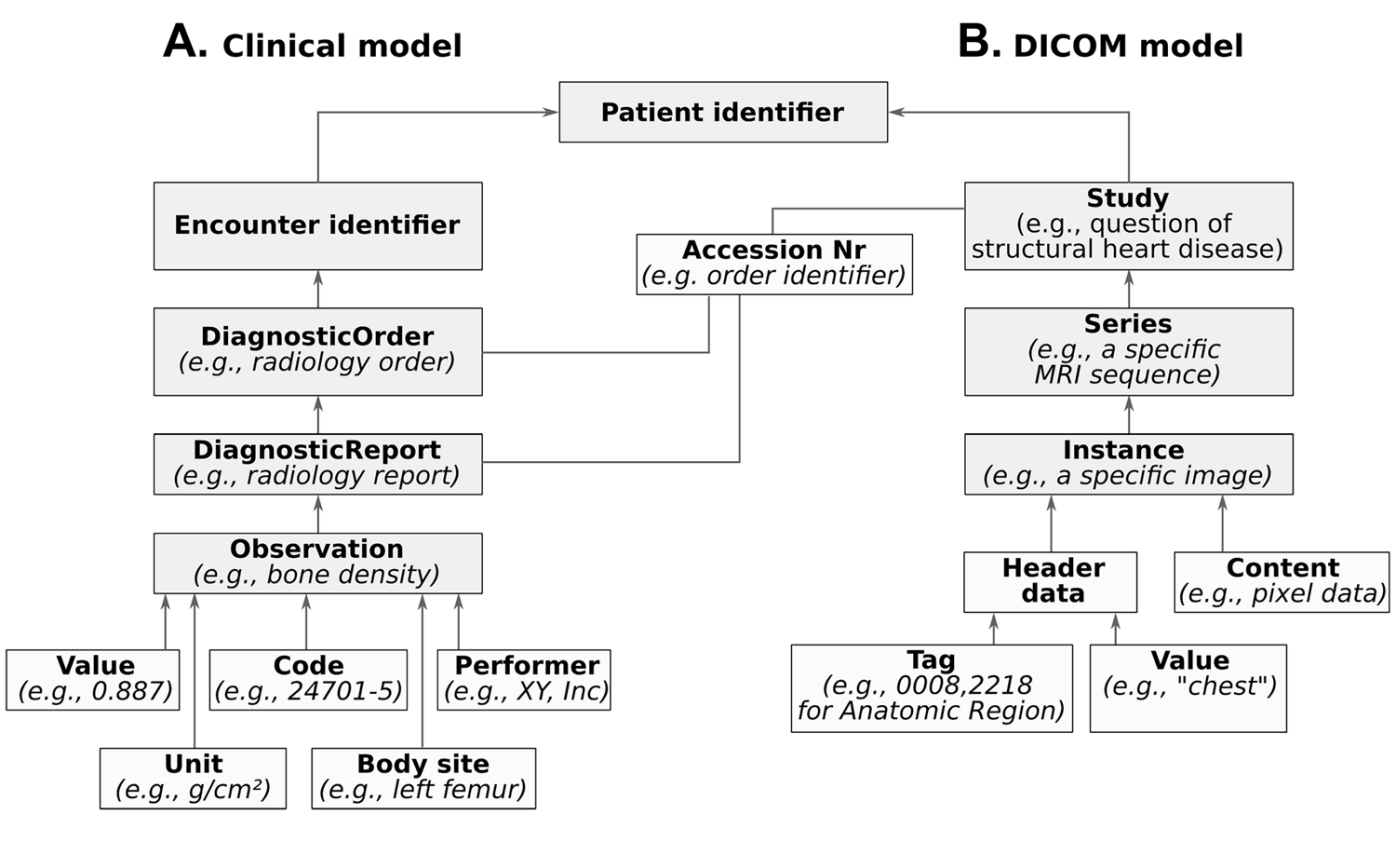
Example of a clinical and DICOM (PACS) data model. Only the links to the next level in the hierarchy are shown. Elements with a gray background structure the data at different levels. (MRI, magnetic resonance imaging)

Hospital information systems (HIS) may consist of many subsystems, each with own specific data models. They may use open standards (e.g., OpenEHR,^2^ FHIR,^3^ IHE^4^) for storage and exchange, or proprietary data models. Each item is connected to a patient with a unique patient identifier and an acquisition time. Commonly, data is also mapped to distinct patient hospitalizations using encounter identifier. Multiple encounters might be further linked using an episode of care (e.g., to link data for the treatment of a specific disease). The data is often stored as document (e.g., a structured form) or in various sub-data models (e.g., OpenEHR archetypes or FHIR resources), which may be linked to a document identifier. A document may further contain data values that are linked on multiple levels, e.g., all values of a table or a table row. Overall, this data compilation is very heterogenous. A simplified data hierarchy is illustrated in Fig. 1A.

#### DICOM Data Model

Images are often stored using DICOM in a PACS. The DICOM data model is hierarchically structured and based on a real radiological examination: a single image (an instance), a series of images (e.g., images of a single MRI sequence), and a study containing all series required for the examination of a specific diagnosis. Often, a DICOM study is linked to a radiology report in a radiology information system (RIS) via the accession number (e.g., an order identifier). Furthermore, a patient identifier can be stored. An illustration of the DICOM data model and its linkage to clinical data is shown in Fig. 1B.

### Clinical Data Warehouse

**Fig. 2:**
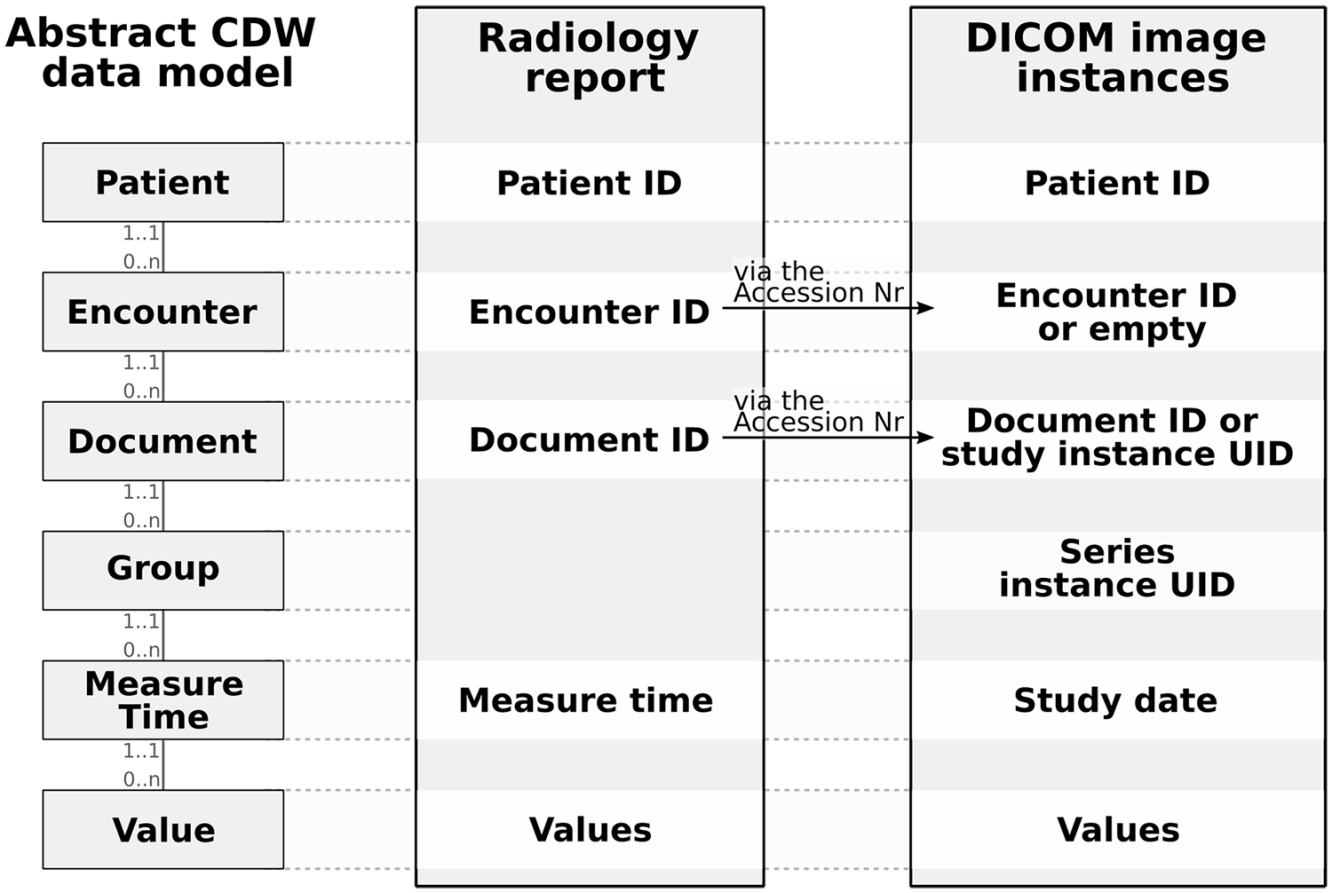
Illustration of the abstract CDW data model (left) and the method we used to map and store parameters of the radiology report (center) and DICOM instances (right). Only connections to the next higher level are shown in the data model. A value can be connected to any level or only to one patient

The CDW contains homogenized and pseudonymized data of a large part of the hospital’s data [29]. Its query system enables constraints on structured data and free text searches in narrative texts (e.g., discharge letters and radiology reports) using regular expressions [30]. Figure 2 illustrates the CDW’s abstract data model in the leftmost column. It is based on an extended EAV model within a relational data base and is similar yet not identical to the star schema of i2b2 [4, 31]. It is derived from the clinical data model described in Fig. 1A and provides the four grouping levels “patient,” “encounter,” “document,” and “group”. Depending on the source data model, values in the CDW are always connected at patient level, almost always at encounter and document level, and sometimes also at group level. Each value is further stored with a concept identifier which links to the value’s metadata.

The main component of the DICOM integration used in this work is a separate middleware (PACS-to-CDW; P2D) that allows pseudonymous querying of a production PACS whose overall architecture was described previously [6]. The middleware essentially accepts the pseudonymous identifiers used in the CDW as input via a REST-style interface, which it maps to the identifiers used in the PACS via an identity management system to perform the most basic DICOM queries C-FIND and C-MOVE (which are most likely available in any production PACS).

## DICOM Data Integration and Query

### DICOM-CDW Integration

Within the CDW, we linked the DICOM imaging data with the clinical data via the radiology report, which are also linked in the clinical domain via the accession number. The report’s data elements were stored with a patient, encounter, and document identifier. Since we need a direct linkage between a report and the DICOM data within the CDW, we adopted these three identifiers to the DICOM data via the accession number. DICOM data originally has a patient identifier, but no encounter and document identifier. Thus, we only have a single grouping level left in the CDW’s data model, which we used to encode the DICOM series via the Series Instance UID. In our experience, this grouping level is of greater interest for research than the single image or the study (potentially containing several modalities and specific examinations).

Some DICOM images may not be associated with an accession number nor a radiology report in the local hospital, e.g., if transmitted from external providers. Such DICOM data was imported with a patient identifier, an empty encounter identifier, and a document identifier that was based on the Study Instance Unique Identifiers (UID). Each value of the DICOM header was stored as separate row within the EAV schema.

### Multilevel Query

We developed a prototypic query system that provides functionality to transform a query defined in a self-developed query language into SQL/Solr queries. The query language allows to constrain the patient selection by filtering the admission date. Most importantly, it allows conditioning of CDW concepts using the concept identifier and various operators (e.g., text contains x, number greater than x) using the operators ‘AND’ and ‘OR’, specifically for each of the available grouping levels. Running a query results in a multilevel collection of data, starting with the filter and an SQL query on the most comprehensive level (i.e., patient) and ending with the most restrictive level (i.e., group). The data selection of an upper group is used as input for the next level. Levels that are not defined are simply skipped, e.g., when querying for a radiology report without a patient/encounter condition, all existing documents of all patients and encounters are selected. Depending on the need, the query results of each level (which may be particularly high at the patient level) or only those of the most restrictive level can be extracted.

**Fig. 3:**
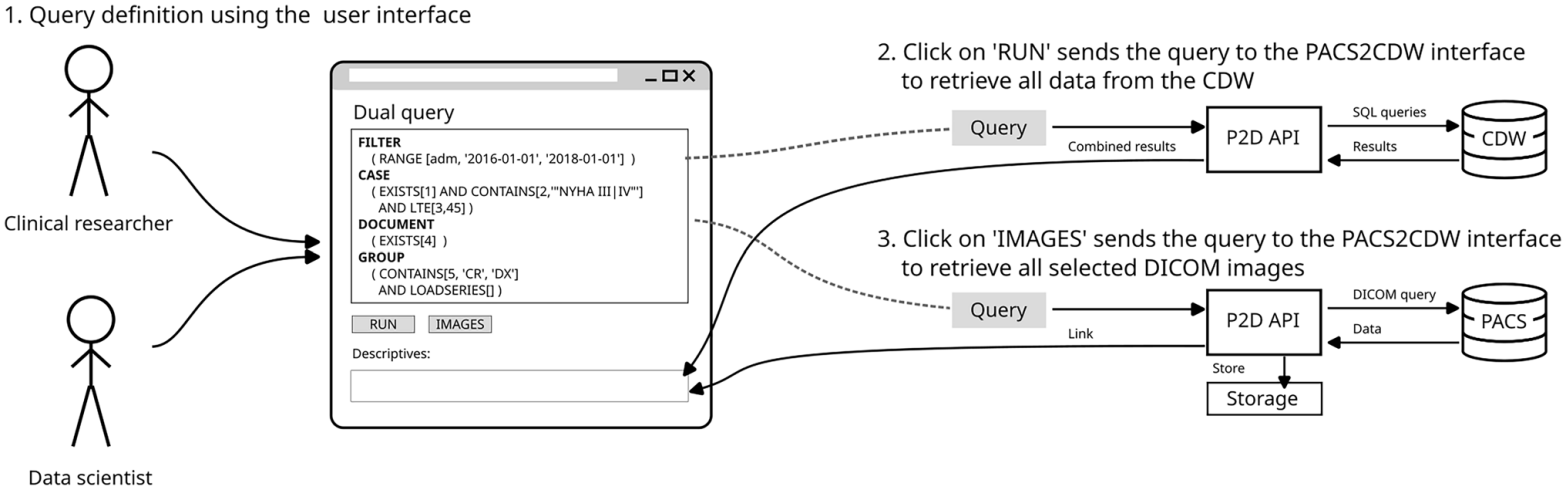
Illustration of the query process: A clinical researcher and data scientist jointly define a query. After execution (2.) the query results are retrieved from the CDW data base. If the query is defined final, the images can be downloaded on another button press (3.)

After entering a query, the system extracts descriptive statistics and a data export of all related data from the CDW by pressing a single button. With a second button press, it extracts all DICOM images of the DICOM series indicated in the query results from the PACS. This process is illustrated in Fig. 3.

### Queries

#### Radiology-Oriented Query

For inquiry #1, we required to search for radiology reports without conditions on patients or encounters. Thus, resulting data sets may contain data from any patient with any disease.

**Fig. 4:**
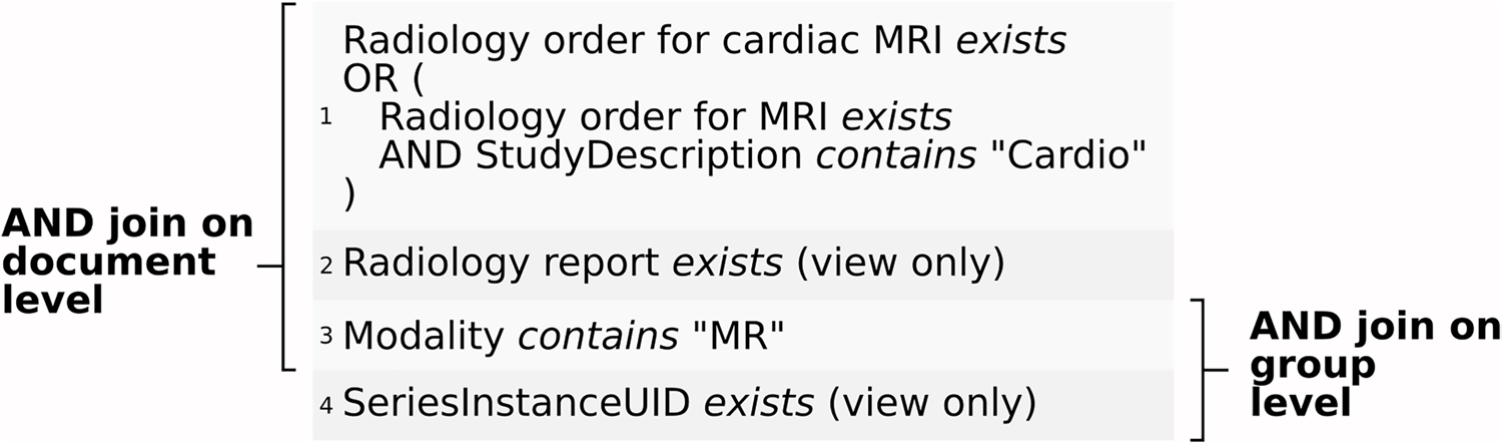
Illustration of the grouping levels we needed to extract data for the first inquiry

We started the query definition on the document level, by selecting a combination of multiple radiology order identifiers defined by a regular expression (each defines a specific radiological examination). A free text search in the DICOM study description may extend the number of resulting documents. On the group level, this data selection is refined to selectively include images acquired by the modality MRI. Figure 4 illustrates the resulting radiology-oriented query.

Since multiple reports may exist per encounter, the report and DICOM parameters needed to be joined on the document level (Fig. 4: 1–3). Of this subgroup, only DICOM series acquired with MRI needed to be queried. Thus, the document level query (Fig. 4: 1–3) needed to be refined to the Series Instance UIDs, joined over the group (Fig. 4: 3–4).

#### Disease-Oriented Query

For inquiry #2, we required to primarily search for patients with the specific condition of having a heart failure.

**Fig. 5:**
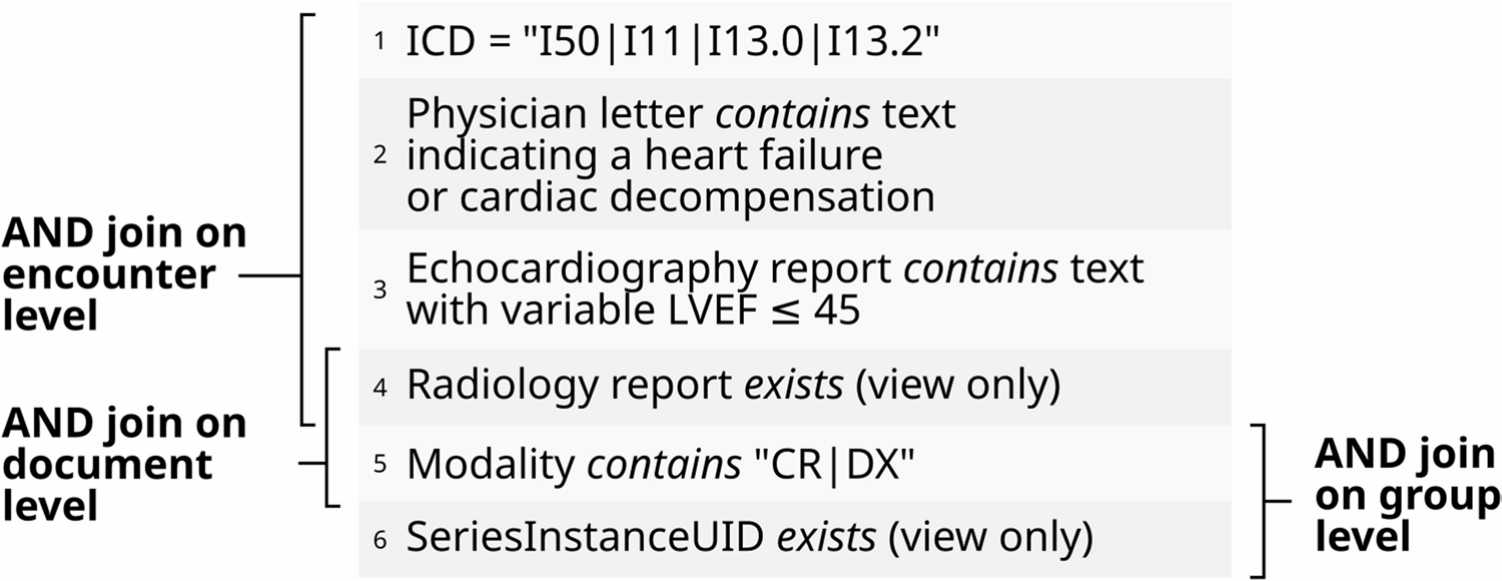
Illustration of the grouping levels we needed to extract data for the second inquiry

We started the query definition on the encounter level by selecting parameters indicative of the disease, following an evaluated heart failure detection algorithm [32]. This algorithm required the search of certain occurrences of free text in discharge letters, conditions on numerical values from echocardiography reports, and the selection of ICD codes. On the document level, we only restrict the data selection to patients with an existing radiology report. Conditions on the group level are similar to the radiology-oriented query. The added complexity is that not only data elements of single documents or a more detailed structure have to be compared to each other, but also data elements of multiple documents of an encounter or a patient. Figure 5 illustrates a disease-oriented query with the conditions from a specific research project.

Most query parameters (clinical or DICOM) originated from different processes, therefore had separate document identifiers, and could only be linked at the patient or encounter level. The clinical values had to be joined on the encounter level, since we are only looking for patients with heart failure and associated images (Fig. 5: 1–4). In contrast, a linkage at the patient level could also reveal images that predate the development of the disease.

## Results

For the two queries, we selected all patient encounters stored in the CDW from the Department of Internal Medicine of the years 2016 and 2017 (“baseline data set”). This data set included 32,153 patients, 64,018 encounters (mean [standard deviation (SD)] of 2546 [629] per month), 31,205 radiology reports (1223 [305] per month), 52,882 DICOM studies (2090 [476] per month), and about 292,369 DICOM series (11,574 [2765] per month). A DICOM study had a mean 5.0 [7.6] series, most often with a single modality (83%), followed by 2 (16%) and 3 (2%) modalities.

### Radiology-Oriented Query

Table 1 presents the descriptive statistics provided by the query interface after performing the query illustrated in Fig. 4. Each row shows metrics of the data subset on the specific grouping level. The query results in 28,651 DICOM series with a mean [SD] 17.6 [6.6] per study. The query includes images from different MRI sequences (that could have been further constrained with additional parameters, e.g., series description, which was not requested by the researchers). Each study was connected to a radiology report.

**Table 1:** Descriptive statistics produced by the query interface described in Fig. 4. The rightmost column presents the number of attributes or unique texts of the query attributes associated with the level

**Level**	**#Patients**	**#Encounter**	**#Documents**	**#Groups**	**#Values**	**#Values per attribute**
Document	2039	2185	2233	49,807	58,738	***Unique:*** Study description = 13 Radiology order = 15 Radiology short finding = 2175 Radiology finding = 1833 Radiology assessment = 2109
Group	1504	1613	1631	28,651	57,302	***Unique:*** Modality = 1 Study instance UID = 1632 Series instance UID = 28,651

This query was joined on two levels in order to arrive at the final result. Assuming we were to join all the data elements of the query at a single level, the query would result in 41,421 (+ 45%) DICOM series when using the document level and 60,701 (+ 112%) DICOM series when using the encounter level. Joining at the document level would lead to the additional selection of the modality PR (presentation state, > 99%); in case of the encounter level, there were 16 additional modalities, mainly PR (43%), XA (X-ray angiography, 25%), CT (computed tomography, 10%), MR (magnetic resonance, 10%), and US (ultrasound, 4%).

### Disease-Oriented Query

Table 2 presents the descriptive statistics produced by the query interface illustrated in Fig. 5. The query resulted in 12,834 DICOM series with a mean [SD] 1,4 [0,5] per study.

**Table 2:** Descriptive statistics provided by the query interface after performing the query described in Fig. 5. The rightmost column presents the number of attributes or unique texts of the query attributes associated with the level. (*LVEF* = left ventricular ejection fraction)

**Level**	**#Patients**	**#Encounter**	**#Documents**	**#Groups**	**#Values**	**#Values per attribute**
Encounter	8185	13,234	14,223	68	38,512	***Unique***: Physician letter = 5125 ***Count***: LVEF = 7759 I11 = 4760 I13 = 71 I50 = 11,638
Document	5181	6650	14,670	1	58,692	***Unique:*** Radiology order = 407 Radiology short finding = 14,245 Radiology finding = 10,443 Radiology assessment = 13,805
Group	4110	5152	9499	12,835	25,670	**Unique:** Modality = 2 Study instance UID = 9529 Series instance UID = 12,834

This query was queried on three grouping levels in order to arrive at correct results, because joining on solely one level would not result in meaningful data since multiple types of documents are required on the encounter level. By contrast, joining data on the most detailed level available at every data entry, i.e., the encounter, such query would result in the same number of encounters and DICOM studies, but would contain 86,346 DICOM series (+ 573%). A query on the encounter level would result in 18 additional modalities, mainly in XA (38%), CT (30%), MR (12%), US (7%), and PR (4%).

## Discussion

We showed the feasibility and benefit of querying combinations of data from medical imaging and clinical subsystems in a CDW based on an EAV data schema. The proposed method integrates DICOM metadata directly into the CDW and allows to reliably obtain quantitative information on the current availability of data with reduced manual steps using a single query (e.g., for feasibility studies). Querying the details of DICOM series in addition to clinical data may be particularly important when imaging data from a large number of patients is required, e.g., for deep learning approaches including both imaging and clinical data. To our knowledge, such a data integration and query capability with constraints at multiple grouping levels in a single query has not been demonstrated before.

In order to provide such a query capability, we advanced an approach that has been started by existing CDWs. CDWs mainly provide the two grouping levels patient and encounter, to which we have added the levels document and group. This allows to integrate data values that may be linked on four levels, which can be used to include small subsets of the original data models, e.g., the DICOM information model. Since the data models of the different data domains differ, the meaning of the grouping layers also differs.

We demonstrated the feasibility of a combined query that we constrained on all four levels for a single sub-model, i.e., DICOM. The execution of the query leads to the provision of descriptive statistics, partitioned into the grouping levels used in the query, e.g., count of patient encounter, radiology reports (document), and DICOM series (group). Another usage is conceivable that requires such constraining on all levels for multiple data domains, e.g., to identify patients with a myocardial infarction based on two variables of a single blood sample (sub-model 1) and specific DICOM series (sub-model 2).

We currently enable the mapping of source data models with up to four levels to the EAV data model. Especially the document and group level could be mapped to any association of data elements that is of interest. However, we do not yet provide a generic solution to allow for more than four levels. We neither provide a fully standardized method to annotate the meaning of the group levels in the CDW with its usage in the data model (e.g., in data domain “imaging,” a group refers to the linkage using the Series Instance UID). Furthermore, we developed an imaging-optimized prototype that can be applied for similarly framed used cases, but is not as easy to use as our standard CDW query interface.

We illustrated, how we were able to improve the accuracy of the presented data using multiple grouping levels compared to queries that only combine data elements on a single level. Accordingly, merging all data elements of the radiology-oriented query at the document level would result in a near doubling of the number of identified DICOM series. Since these were almost exclusively of type PR (presentation state), this would not have significantly affected the image download from the PACS, but might have distorted the display of the correct number of series. In any case, the impact of using multiple grouping levels on the numbers presented at the encounter level would be large.

We integrated data into the CDW as provided by the DICOM data model and then constrained it while querying. An alternative to the integration of granular data to the CDW is to them as pre-aggregated data. However, such strategy would impede a quick usage for new usage scenarios. Furthermore, since duplication of a complete PACS is not practical, we would have to access the production PACS each time a new variable of interest is being defined.

We showed queries on DICOM parameters that were retrieved via DICOM C-FIND. While an extraction of all DICOM parameters using C-MOVE would be possible, it would require the transfer of at least one image from each DICOM series. Since this might result in a workload that impacts on the performance of the clinical systems or demands much more time, we only did this when required for subsets of patients yet. A quicker access to all DICOM metadata would be the usage of a PACS that provides web-based parts of the DICOM standard, e.g., QIDO and WADO.^5^ However, not every PACS supports these nor might a hospital department easily acquire such an extension for research purpose. Even then, metadata extraction from a pre-filled CDW would still be faster for large queries. The direct access to the internal data structure of a PACS would provide the fastest solution to access DICOM files of a PACS [28], but could provide a major vulnerability for a production PACS.

The solution presented here is most interesting for CDWs that are based on an EAV data schema. The CDW that is probably most often used is i2b2 [15], which provides an EAV schema that allows storing values with a patient, an encounter, and a modifier. The latter could be used similarly to one of the levels “document” or “group,” i.e., to providing not only a single value per concept, but also its complementary attributes. In practice, however, the i2b2 query user interface usually queries on the patient or encounter level.^6^

An alternative would be the usage of common data models (CDM) that separate data domains into specific database tables. The OMOP-CDM^7^ for example does not provide a single table for all values, but provides a multitude of tables each with a special purpose, and consists of combinations of relational and EAV data schemas. The fastest way to include DICOM data might be the usage of an existing table (e.g., “measurement” or “observation,” both with an EAV schema). However, the OMOP-CDM only links a value to a patient or an encounter. Importantly, a document or group in terms of a collection of parameters measured from a single specimen at a specific time is not provided.^8^ Thus, it might be more straightforward to extend the CDM with a specific table for DICOM data.

If we were to follow this reasoning from an a single EAV table to domain-specific tables further, we could also use the data models of HL7 FHIR or OpenEHR itself, as suggested by Paff et al. [33]. This would allow to document even more granular data including links between data values. Multiple values of a laboratory panel for example would be documented within the FHIR resource ‘DiagnosticReport’. However, this approach has the disadvantage of many different sub-data models thus requiring multiple small queries.

With “Dr. Warehouse,” Garcelon et al. [34] developed a CDW that centers on documents in their database schema. This allows to store documents as text, in combination to extracted parameters. The CDW then can be searched in relation to the document, the encounter or patient level also providing a free text search option to query all documents. Despite this extensive search functionality in the graphical user interface, however, it does not seem to provide possibilities to constrain multiple variables of a single document yet.

Given a variety of base data models being integrated into CDWs and research data models, and a concomitant increase in data complexity, an ideal solution might be to provide a generic query interface for a general unrestricted CDW query and a domain-specific query interface for specific used cases that guides through a query as described by Horvath et al. [35].

We are not aware of a CDW system that integrates both clinical data and data from imaging systems themselves, but rather only data derived from image data, such as radiology reports. Instead, many CDWs offer an image store for manual image upload or semi-manual PACS download and are not optimized for access to all PACS data, as described in the introduction. The CDW system closest to the one we propose is the i2b2 PACS integration, which lacks metadata integration and offers multistage querying of a production PACS [15]. They provided a special module to i2b2 that allows for various query constraints based on a list of patients, e.g., obtained from the standard i2b2 query interface. The query is directly sent to the DICOM interface of the connected PACS. Consequently, their approach mandates multiple query steps, which heavily slows speed of result generation when querying a larger group of patients.

As opposed to a single CDW system containing clinical and imaging data, a viable path could also be to virtually integrate separate imaging-oriented warehouse systems (e.g., Dicoogle) and clinical systems (i.e., i2b2), e.g., using a federated query system. Dicoogle is a comprehensive PACS developed for research purposes that enables distributed queries across multiple PACS sites. It creates indices of metadata for a fast query capability and allows for many plugins and applications on image-oriented studies [28, 36]. It is not directly used with a CDW and its query is image-centric [28].

We added metadata from DICOM files to the CDW. This is relatively simple, but can support many used cases [14]. A major goal remaining is to also integrate variables of features extracted from the imaging raw data, i.e., the pixel data. Several content-based image retrieval solutions in the context of dedicated image-oriented CDW and analysis systems have been developed [37–42]. However, such systems have not been comprehensively integrated into a productive hospital CDW, which remains an open task.

## Conclusion

An EAV data model can be extended to store data from clinical systems and PACS on multiple levels to enable combined querying with a single query to quickly display actual frequency data.

## Footnotes

1 ICD (International Statistical Classification of Diseases and Related Health Problems).

2 OpenEHR Information Model, https://specifications.openehr.org/releases/RM/latest/ehr.html (accessed 2022–02-14).

3 HL7 FHIR, Administration Module, http://www.hl7.org/fhir/administration-module.html (accessed 2022–02-14).

4 Integrating the Healthcare Enterprise, IT Infrastructure (ITI) Technical Framework Volume 2 (ITI-22) and Technical Framework Volume 3 (ITI TF-3).

5 The DICOM standard contains parts for web-based access. Query based on ID for DICOM Objects (QIDO) and Web Access to DICOM Objects (WADO) are the most interesting for this project. Details see https://www.dicomstandard.org/ (accessed 2022–10-20).

6 i2b2 Web Client Query Tool. https://community.i2b2.org/wiki/display/webclient/3.+Query+Tool (accessed 2021–07-20).

7 OMOP Common Data Model. https://ohdsi.github.io/TheBookOfOhdsi/CommonDataModel.html (accessed 2021–07-20).

8 OMOP Wiki. https://www.ohdsi.org/web/wiki/doku.php (accessed 2021–07-20).
